# High‐cell‐density fed‐batch strategy to manufacture tailor‐made P(HB‐ *co* ‐HHx) by engineered *Ralstonia eutropha* at laboratory scale and pilot scale

**DOI:** 10.1111/1751-7915.14488

**Published:** 2024-06-08

**Authors:** Isabel Thiele, Lara Santolin, Svea Detels, Riccardo Osele, Peter Neubauer, Sebastian L. Riedel

**Affiliations:** ^1^ Chair of Bioprocess Engineering, Institute of Biotechnology Technische Universität Berlin Berlin Germany; ^2^ Department of Biotechnology University of Verona Verona Italy; ^3^ Environmental and Bioprocess Engineering Laboratory, Department VIII – Mechanical Engineering, Event Technology and Process Engineering Berliner Hochschule für Technik Berlin Germany

## Abstract

The transition towards a sustainable bioeconomy requires the development of highly efficient bioprocesses that enable the production of bulk materials at a competitive price. This is particularly crucial for driving the commercialization of polyhydroxyalkanoates (PHAs) as biobased and biodegradable plastic substitutes. Among these, the copolymer poly(hydroxybutyrate‐*co*‐hydroxyhexanoate) (P(HB‐*co*‐HHx)) shows excellent material properties that can be tuned by regulating its monomer composition. In this study, we developed a high‐cell‐density fed‐batch strategy using mixtures of fructose and canola oil to modulate the molar composition of P(HB‐*co*‐HHx) produced by *Ralstonia eutropha* Re2058/pCB113 at 1‐L laboratory scale up to 150‐L pilot scale. With cell densities >100 g L^−1^ containing 70–80 wt% of PHA with tunable HHx contents in the range of 9.0–14.6 mol% and productivities of up to 1.5 g L^−1^ h^−1^, we demonstrate the tailor‐made production of P(HB‐*co*‐HHx) at an industrially relevant scale. Ultimately, this strategy enables the production of PHA bioplastics with defined material properties on the kilogram scale, which is often required for testing and adapting manufacturing processes to target diverse applications.

## Graphical abstract

Bioprocess development for the production of polyhydroxyalkanoate (PHA) biopolymers with tailored monomer compositions from 1st‐generation feedstocks: Fed‐batch strategies for production at high cell densities with a productivity of up to 1.5 g_PHA_ L^−1^ h^−1^ from laboratory scale to pilot scale will be demonstrated.

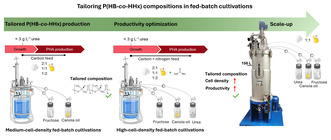

## INTRODUCTION

In 2008, the EU implemented the bioeconomy strategy, emphasizing the global necessity to shift from petroleum‐based industries to biobased, circular production concepts. In line with this transition is the commercialization of polyhydroxyalkanoate (PHA) bioplastics that can be produced by microbial fermentation from diverse renewable raw materials and waste streams (Riedel & Brigham, 2020) and are fully biodegraded after disposal in common natural environments (Laycock et al., 2020). However, efficient production processes are imperative to compete with low‐cost fossil‐based plastics.

PHAs are produced by numerous prokaryotic species, including *Ralstonia eutropha* (also known as *Cupriavidus necator*), as intracellular carbon and energy storage compounds. The wild type of *R. eutropha* has the remarkable natural ability to accumulate up to 90% of its cell dry weight (CDW) as the PHA homopolymer polyhydroxybutyrate (PHB) and has been genetically engineered to utilize a broad portfolio of feedstocks and produce various PHA copolymers with versatile properties (Sohn et al., 2021; Volodina et al., 2016). Material properties of PHAs depend strongly on their monomer composition, which are classified into *short‐chain‐length* (*scl*, 4–5 carbon atoms) or *medium‐chain‐length* (*mcl*, 6–14 carbon atoms) monomers (Rehm, 2003).

Taking advantage of its natural ability to utilize oleaginous feedstocks that ensure high PHA conversion at low dilution (Gutschmann et al., 2023), *R. eutropha* Re2058/pCB113 was introduced with a heterologous PhaC synthase that enables the incorporation of HHx monomers from β‐oxidation intermediates, resulting in the *scl‐mcl* copolymer poly(hydroxybutyrate‐*co*‐hydroxyhexanoate) (P(HB‐*co*‐HHx)) (Budde et al., 2011). This strain synthesizes P(HB‐*co*‐HHx) from lipids but can only produce the *scl*‐PHA PHB or poly(hydroxybutyrate‐*co*‐hydroxyvalerate) from sugars, alcohols, or respective short‐chain carboxylic acids (Riedel & Brigham, 2019), offering a strategic advantage: By employing mixtures of fructose and an oleaginous feedstock, the HHx monomer content can be precisely adjusted, with the maximum achievable HHx content dictated by the oily substrate. Previously, we reported a simple batch strategy to produce P(HB‐*co*‐HHx) with controlled molar HHx content in the range of 2–17 mol% HHx in 1‐L laboratory‐scale bioreactors using mixtures of fructose and canola oil (Santolin et al., 2023). Control of the HHx monomer content by substrate mixtures of other feedstocks such as date seed oil, date molasses, or palm kernel oil had also been reported by other groups (Murugan et al., 2016; Purama et al., 2018).

Our group and others showed that by controlling the HHx monomer content of P(HB‐*co*‐HHx), its processing window and application range can be adjusted, impacting also crystallinity and elongation at break (Arikawa & Sato, 2022; Kehail et al., 2015; Miyahara et al., 2020; Noda et al., 2005; Thiele et al., 2024). Along with diverse material properties, PHAs find versatile applications in packaging, biomedicine, and non‐woven fabrics (Mahato et al., 2023). However, despite emulating the features of the seven top‐selling fossil plastics accounting for an annual production of 230 million tonnes, the installed PHA manufacturing capacity in 2021 was estimated at only 48 kt/annum (Mukherjee & Koller, 2023; Ravenstijn, 2022).

High production costs related to expensive raw materials and complex downstream processes combined with the inherent challenge of biomanufacturing to maintain consistent product quality are the main factors hindering the commercialization of PHAs (Che et al., 2023). To ensure competitive market prices, optimized cultivation strategies, such as fed‐batch cultivations, which are substrate‐flexible and deliver increased productivities with reliable polymer quality, are required. Fed‐batch processes with high productivities >2 g_PHA_ L^−1^ h^−1^ using other recombinant strains fed with single substrates or oils combined with fatty acids have been previously reported (Arikawa & Matsumoto, 2016; Sato et al., 2013, 2015). Nevertheless, there is no mixed substrate strategy to precisely control the resulting copolymer composition reported at high‐cell densities at an industrially relevant scale.

In this study, we extend our fructose and canola oil feeding strategy to high‐cell‐density fed‐batch cultivations of *R. eutropha* Re2058/pCB113 at 1‐L laboratory scale to 150‐L pilot scale, achieving productivities up to 1.5 g_PHA_ L^−1^ h^−1^ while maintaining predictable PHA compositions. This strategy allows the production of tailor‐made PHAs with defined properties in the kilogram scale for various applications.

## EXPERIMENTAL PROCEDURES

### Bacterial strain

Recombinant *Ralstonia eutropha* Re2058/pCB113 was used in all experiments (Budde et al., 2011). The strain was stored in 20% (v v^−1^) glycerol at −80°C.

### Seed train and media compositions

Tryptic soy broth (TSB) media, agar plates, and mineral salt media (MSM) compositions have been described previously (Gutschmann et al., 2019). A first preculture in TSB media was carried out as described by Santolin et al. (2023). A second preculture in MSM was performed as described by Santolin et al. (2021) and incubated for 24 h at 30°C and 200 rpm. Mixtures of fructose and canola oil (Edeka Zentrale AG and Co. KG, Germany) were used as carbon sources, and urea was used as the sole nitrogen source in the MSM. Precise mixture ratios and total carbon and nitrogen concentrations (g L^−1^) are specified in the text according to calculations described previously (Santolin et al., 2023). A final carbon‐to‐nitrogen ratio of 22 (C/N, g g^−1^) was applied to all cultivations. Fructose, canola oil, and urea concentrations in the second preculture always equalled initial bioreactor concentrations. During bioreactor runs, after 24 h of cultivation MgSO_4_, CaCl_2_, K_2_SO_4_, and trace elements were added to initial concentrations to avoid limitation of these nutrients. All chemicals were purchased from Carl Roth GmbH and Co. KG, Germany, unless stated otherwise.

### Laboratory scale bioreactor cultivations

**FIGURE 1:**
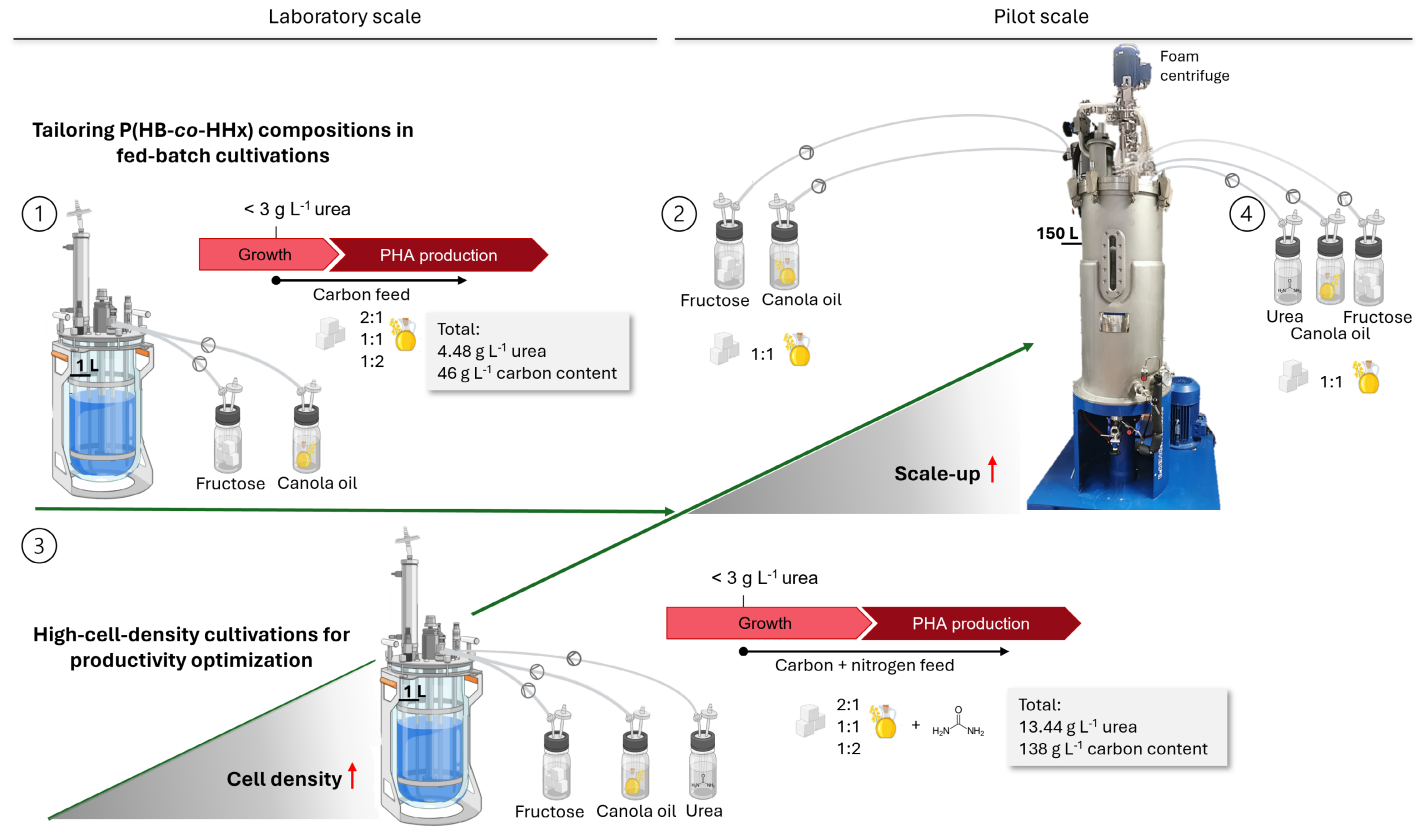
Schematic overview of the development, optimization, and scale‐up of the cultivation strategy. (1) 1‐L fed‐batch cultivations with *Ralstonia eutropha* Re2058/pCB113 using 2:1; 1:1, and 1:2 fructose and canola oil mixtures at a final carbon content of 46 g L^−1^ and 4.48 g L^−1^ urea as nitrogen source. (2) Scale‐up of the fed‐batch strategy to 150‐L pilot scale (working volume 100 L) using the 1:1 fructose and canola oil mixture. (3) Optimization of the strategy to higher cell densities by supplementation of urea to a final concentration of 13.44 g L^−1^ and increased final carbon content to 138 g L^−1^ with 2:1, 1:1, and 1:2 fructose and canola oil mixtures. (4) Scale‐up of the high‐cell‐density strategy to pilot scale using the 1:1 fructose and canola oil mixture.

Fed‐batch strategies were developed in 1‐L parallel Multifors 2 bioreactors (Infors AG, Switzerland) equipped with two six‐blade Rushton impellers (Figure 1). Temperature was kept constant at 30 ± 0.1°C, and pH was maintained at 6.8 ± 0.1 through controlled addition of 1 M H_3_PO_4_ and 2 M NaOH. 200 rpm and 0.5 vvm were set as initial process parameters, and dissolved oxygen (DO) was prevented from dropping below 40% by an automated cascade increasing stirrer speed up to 1500 rpm followed by the increment of aeration up to 1 vvm, if necessary. Foam was mechanically broken as described previously (Riedel et al., 2012), and if needed, antifoam agent (Nol‐LG126; Adeka) was added to the culture (max. final amount: 1 mL). Cultivations were carried out with a starting volume of 450 mL MSM that was inoculated with 50 mL of the second preculture. Cultivations were always run for 72 h and performed in biological duplicates.

#### Medium‐cell‐density fed‐batch cultivations

Three different mixtures of fructose and canola oil, namely 2:1, 1:1, and 0.5:1 (carbon ratio of fructose to canola oil in g g^−1^), were applied in an early fed‐batch approach to reach approximately 40 g L^−1^ CDW with tailored P(HB‐*co*‐HHx) compositions. Initial batch media contained 4.48 g L^−1^ urea and 10 g L^−1^ carbon of the correspondent fructose and canola oil mixture. When urea concentrations dropped below 2–3 g L^−1^, pure canola oil and 50% (w v^−1^) fructose were fed constantly at 0.6 mL h^−1^ and 2 mL h^−1^ until yielding a final carbon content of 46 g L^−1^ of the correspondent mixture.

#### High‐cell‐density fed‐batch cultivations

The fed‐batch approach was further optimized to reach high cell densities in the range of 120 g L^−1^ CDW. Again, 2:1, 1:1, and 0.5:1 fructose and canola oil mixtures were applied to achieve tailored P(HB‐*co*‐HHx) compositions (Figure 1). Initial batch phase conditions (10 g L^−1^ carbon fructose and canola oil mixture and 4.48 g L^−1^ urea) were maintained as in previous experiments, but the carbon amount fed during the fed‐batch phase was tripled to yield a final amount of 138 g L^−1^, and additionally, urea was supplemented to a final total concentration of 13.44 g L^−1^. When urea concentrations dropped below 2–3 g L^−1^ a 18.34% (w v^−1^), urea solution was fed at an initial rate of 2 mL h^−1^, and pure canola oil and 50% (w v^−1^) fructose solution were fed at an initial rate yielding 1 g h^−1^ carbon of the correspondent fructose‐to‐canola oil ratio. After 2 h, all feeding rates were doubled, and 6 h later, the initial fructose and canola oil feeding rates were shortly tripled until nitrogen depletion set in. During nitrogen limitation, the feeding rates were lowered to the previous doubled rate until the complete carbon source was fed. Urea and fructose concentrations were measured in each sample during the cultivation, and feeding rates were adjusted correspondingly to avoid depletion or high accumulation of the substrates. Detailed feeding regimes applied for each cultivation can be withdrawn from the results section.

### Pilot scale bioreactor cultivations

The seed train for pilot scale cultivation was slightly different to the laboratory scale cultivations. A first preculture consisting of 50 mL TSB media inoculated with 5 colonies from an agar plate was incubated in 250‐mL Ultra Yield Flasks (Thomson Instrument Company, USA) covered with an AirOtop membrane (Thomson Instrument Company, USA) for 15 h at 200 rpm and 30°C until reaching an OD_600_ of 5. The second preculture was carried out in a 6.6‐L laboratory scale bioreactor (BIOSTAT Aplus, Sartorius AG, Germany) containing 4.95 L MSM that was inoculated with the 50 mL of the first preculture. The bioreactor was aerated with 0.5 vvm, and temperature was kept at 30 ± 0.1°C during the 24 h of preculture cultivation. The DO was maintained above 40% using a stirrer cascade between 200 and 950 rpm.

The pilot scale cultivations were carried out in a 150‐L bioreactor (P150; Bioengineering AG, Switzerland) equipped with three, 6‐blade Rushton impellers. The culture was initially aerated with 0.5 vvm and mixed with an initial stirrer speed of 150 rpm. The stirrer speed was manually increased in 50 rpm steps throughout the cultivations to prevent oxygen limitation. Air supply was reduced stepwise towards the end of the cultivation to prevent strong foaming. The temperature was kept constant at 30 ± 0.1°C, and pH was maintained at 6.8 ± 0.2 through controlled addition of 6 M NaOH and 3 M H_3_PO_4_. The pilot scale bioreactor was equipped with an external foam centrifuge Foamex 10 P (Frings, Germany) that prevented foam from reaching the off‐gas filter.

#### Pilot scale medium‐cell‐density fed‐batch cultivations

The medium‐cell‐density fed‐batch strategy of the 1:1 fructose and canola oil mixture was scaled up to pilot scale. The bioreactor had an initial working volume of 85 L (80 L MSM inoculated with 5 L of the second preculture) (Figure 1). Mimicking the laboratory scale, the MSM initially contained 4.48 g L^−1^ urea and 10 g L^−1^ carbon mixture. Once urea concentrations below 2–3 g L^−1^ were measured, pure canola oil and 50% (w v^−1^) fructose were fed constantly at 0.1 L h^−1^ and 0.34 L h^−1^, respectively. 21 h later, the feed rates were halved to avoid accumulation of fructose. The final carbon content provided during the batch and fed‐batch phases yielded 46 g L^−1^.

#### Pilot scale high‐cell‐density fed‐batch cultivations

As a proof of concept, the high‐cell‐density fed‐batch strategy with the 1:1 fructose and canola oil mixture was finally transferred to pilot scale. The initial working volume was reduced to 65 L (60 L MSM + 5 L preculture) (Figure 1). Initial batch conditions were maintained as in all other cultivations (4.48 g L^−1^ urea and 10 g L^−1^ carbon mixture), and after urea concentrations dropped below 2–3 g L^−1^, the fed‐batch phase started by feeding 85 mL h^−1^ of a 55% (w v^−1^) urea solution, 90 mL h^−1^ pure oil, and 270 mL h^−1^ of a 60% (w v^−1^) fructose solution. All feed rates were doubled after 7 h, and 24 h later, fructose and canola oil feeding rates were again lowered to the initial setpoints. The final carbon content provided during the batch and fed‐batch phases yielded 138 g L^−1^, and the total urea concentration provided added up to 13.44 g L^−1^.

### Analytical methods

During the complete course of the cultivations, 5–15 mL aliquots were sampled from the bioreactors in pre‐weighed 15‐mL centrifuge tubes for CDW determination. The samples were centrifuged for 10 min at 4°C and 8000 × *g*, and pellets were washed with 3.5 mL ice‐cold deionized water and 1.5 mL ice‐cold *n*‐hexane to remove media components and residual oils. The washed pellets were suspended in approximately 2 mL deionized water and frozen at −80°C prior to lyophilization (Gamma 1–20; Martin Christ Gefriertrocknungsanlagen GmbH, Germany).

The PHA content and composition of the dried cells were determined using a methanolysis protocol for sample preparation and gas chromatography with a flame ionization detector as previously described (Bartels et al., 2020).

Fructose concentration in the supernatant was measured via HPLC‐RID (HPLC‐RID 1200 series; Agilent Technologies, USA) with an Agilent Hi‐Plex Ca column and 20 μL injection volume at a flux of 0.6 mL min^−1^ for 62 min at 80°C using DI H_2_O as the eluent. To prevent clogging of the column, the supernatant was washed 3 times in a rotary overhead shaker (Rotator Drive STR4; Stuart Scientific, Cole‐Parmer, Germany) for 15 min using equal volumes of *n*‐hexane and water to remove residual oil. Subsequently, the aqueous phase was heat‐treated at 85°C for 5 min to precipitate proteins that were separated by centrifugation at 21,500 × *g* for 2 min at 4°C. The supernatant was then filtered into the HPLC vial through a 0.2‐μm nylon syringe filter. Urea concentration in the supernatant was determined photometrically using an enzymatic assay (Megazyme Ltd., Bray, Ireland) according to the manufacturer's instructions adapted to 1‐mL cuvettes.

## RESULTS

### Tailoring P(HB‐*co*‐HHx) compositions in fed‐batch cultivations

The first aim of this study was to evaluate whether the previously developed substrate mixture batch strategy to tailor molar compositions of P(HB‐*co*‐HHx) (Santolin et al., 2023) was applicable to a fed‐batch process.

#### Laboratory scale

**FIGURE 2:**
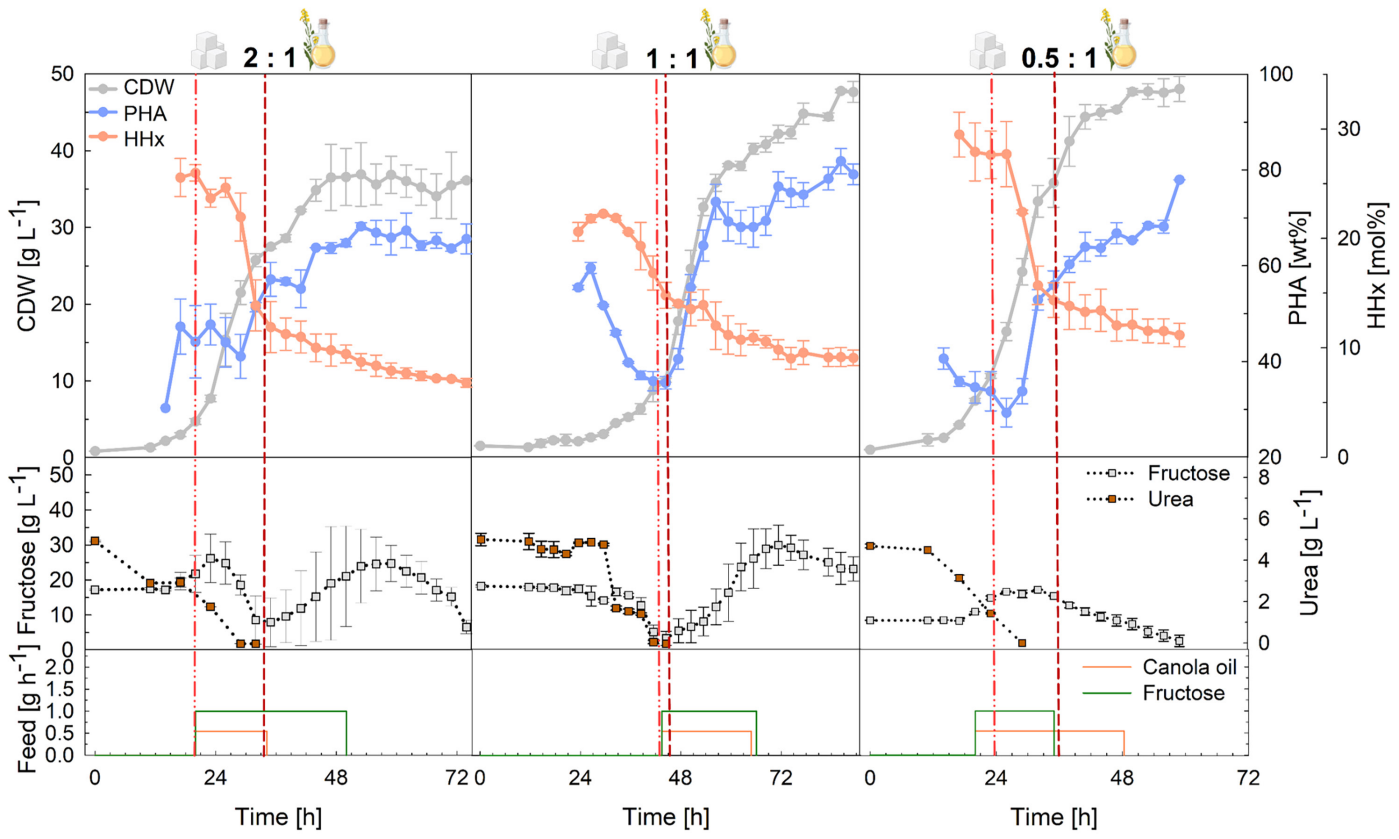
Tailored P(HB‐*co*‐HHx) production in fed‐batch mode at laboratory scale using *R. eutropha* Re2058/pCB113 with mixtures of fructose and canola oil as carbon sources and urea as nitrogen source. Cell dry weight (CDW, g L^−1^), PHA content of CDW (PHA, wt%), HHx content of PHA (HHx, mol%), fructose concentration (g L^−1^), urea concentration (urea, g L^−1^), and fructose and canola oil feeding rates (feed, g h^−1^) over the whole process time are shown. The ratio of fructose to canola oil (g g^−1^) is given for each substrate mixture. The final carbon content of all cultivations was 46 g L^−1^, and a C/N ratio of 22 g g^−1^ was used. Error bars represent the standard deviation of biological replicates. Dashed lines indicate the feeding start (light red, dash–dot–dot) and nitrogen limitation (dark red, short dash).

Three fructose‐to‐canola oil ratios (2:1, 1:1, and 0.5:1) were evaluated in duplicate 1‐L laboratory scale fed‐batch cultivations with the aim of obtaining about 40 g L^−1^ CDW with tailored P(HB‐*co*‐HHx) compositions (Figure 2). The cultivation with 1:1 fructose and canola oil mixture showed a longer lag phase, showing a higher PHA content at inoculation, so the cultivation was extended to 86 h in contrast to the others carried out for 72 h. Over the course of the cultivation, the HHx content decreased in all cultivations, approaching the expected trend of 6.8, 9.1, and 11.2 mol% at 64, 86, and 45 h from the lowest to the highest canola oil ratio, respectively. With the 1:1 and 0.5:1 fructose‐to‐canola oil mixtures, higher biomass yields of almost 50 g L^−1^ were reached, while with the highest fructose ratio in the 2:1 mixture, PHA accumulated only to 57.0 wt% reaching 36.1 g L^−1^ CDW. In accordance with this, 20 g L^−1^ fructose remained unconsumed for this mixture ratio at the end of the cultivation. Disregarding the longer lag phase of the 1:1 cultivation, the process was comparably shorter with higher oil contents.

#### Pilot scale

**FIGURE 3:**
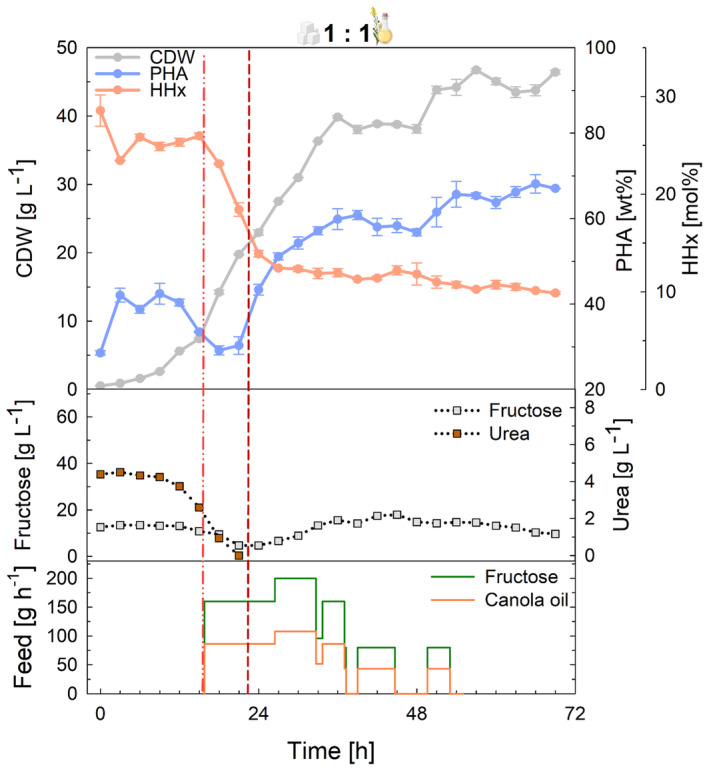
Tailored P(HB‐*co*‐HHx) production in fed‐batch mode at 150‐L pilot scale using *R. eutropha* Re2058/pCB113 with a 1:1 mixture of fructose and canola oil as carbon sources and urea as nitrogen source. Cell dry weight (CDW, g L^−1^), PHA content of CDW (PHA, wt%), HHx content of PHA (HHx, mol%), fructose concentration (g L^−1^), urea concentration (urea, g L^−1^), and fructose and canola oil feeding rates (feed, g h^−1^) over the whole process time are shown. The final carbon content of the cultivation was 46 g L^−1^, and a C/N ratio of 22 g g^−1^ was used. Error bars represent the standard deviation of technical duplicates. Dashed lines indicate the feeding start (light red, dash–dot–dot) and nitrogen limitation (dark red, short dash).

After successfully transferring the substrate mixture strategy to control P(HB‐*co*‐HHx) compositions to fed‐batch cultivations, the process was scaled up to a 150‐L pilot scale fermenter with 100 L working volume applying the 1:1 mixture (Figure 3). At pilot scale, more accurate pumps and control of the feeding rates led to lower accumulation of fructose during the cultivation, as the pumps were not working on their lower limit (like in the laboratory scale), and thus, a continuous feed could be conducted according to plan, with a final residual amount of approximately 10 g L^−1^. Moreover, the lag phase was considerably shortened as the second preculture was performed in a bioreactor, and in comparison, nitrogen was depleted sooner than in any of the laboratory‐scale cultivations. The HHx fraction prior to nitrogen limitation was slightly higher than in 1‐L scale but decreased to the desired amount of about 10 mol% HHx over the cultivation, showing the scalability of the strategy. A final CDW of 46.4 g L^−1^ containing 67.1 wt% PHA agreed with the previous laboratory‐scale results. During the scale‐up, the feed was decreased during the production phase as consumption rates slowed down and accumulation of fructose was to be prevented.

### Optimization of fed‐batch strategy to high‐cell‐density cultivations

To further increase cell densities while maintaining high PHA contents with tailored compositions and thereby raising productivities, the total carbon content supplied was increased to 138 g L^−1^ and the growth phase was prolonged by feeding urea to a final concentration of 13.44 g L^−1^. Urea and fructose concentrations were measured *offline* in real time (with a measurement delay of 40 min and 2 h, respectively) to adapt feeding rates and prevent accumulation, limitation, or depletion of the substrates. Even though the amount of active biomass was increased above 30 g L^−1^, no oxygen limitation was faced even at pilot scale.

#### Laboratory scale

**FIGURE 4:**
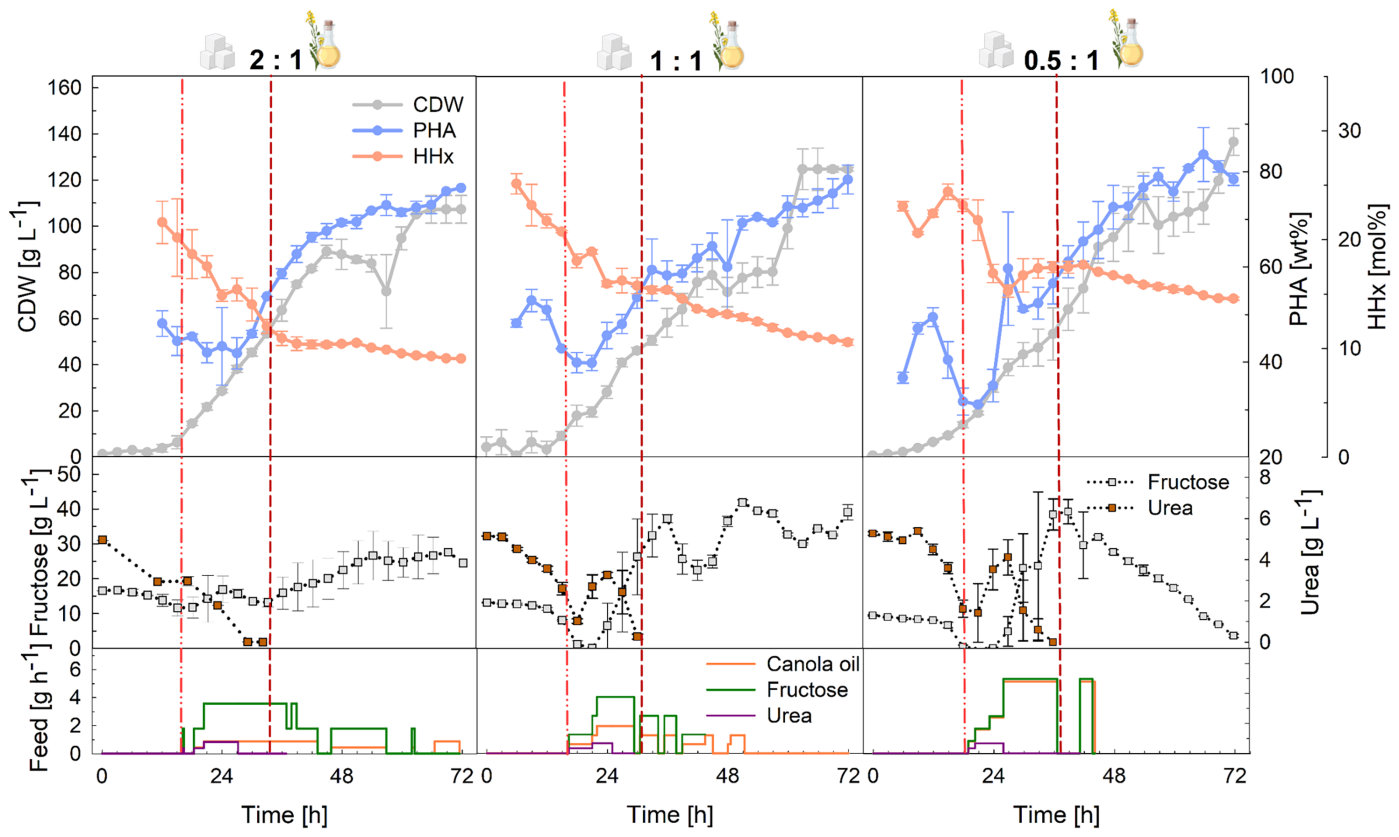
Tailored P(HB‐*co*‐HHx) production in high‐cell‐density fed‐batch mode at laboratory scale using *R. eutropha* Re2058/pCB113 with mixtures of fructose and canola oil as carbon sources and urea as nitrogen source. Cell dry weight (CDW, g L^−1^), PHA content of CDW (PHA, wt%), HHx content of PHA (HHx, mol%), fructose concentration (g L^−1^), urea concentration (urea, g L^−1^), and fructose, canola oil, and urea feeding rates (feed, g h^−1^) over the whole process time are shown. The ratio of fructose to canola oil (g g^−1^) is given for each substrate mixture. The final carbon content of all cultivations was 138 g L^−1^, and a C/N ratio of 22 g g^−1^ was used. Error bars represent the standard deviation of biological replicates. Dashed lines indicate the feeding start (light red, dash–dot–dot) and nitrogen limitation (dark red, short dash).

Again, three different ratios of fructose to canola oil (2:1, 1:1, and 0.5:1) were evaluated in duplicate fed‐batch cultivations in 1‐L laboratory bioreactors (Figure 4). Pumps had to be switched off regularly to prevent the accumulation of substrates, while higher oil contents turned out to be less sensitive and easier to control. Fructose accumulated up to 40 g L^−1^ during the production phase, when the metabolism of the cells slowed down, but this did not have any visible effect on the cells that could further consume the substrate. In the cultivations with 2:1 and 1:1 fructose and canola oil ratios, PHA contents did not increase above 73.0 wt% and residual fructose concentrations of 24.4 and 37.7 g L^−1^ remained unconsumed at the end of the cultivations with about 30 and 23 g L^−1^ of fructose not being fed anymore due to accumulation, respectively. During the growth phase, PHA accumulation was observed in all cultivations already when urea concentrations dropped below 2–3 g L^−1^, the threshold value for the start of feeding (light red dashed line). Final CDWs of 107.3, 124.9, and 136.6 g L^−1^ with 70.7, 73.0, and 78.4 wt% PHAs were reached containing 9.0, 10.6, and 14.6 mol% HHx after 72 h of cultivation, from lower to higher canola oil ratios. The process had productivities of ~1.5 g_PHA_ L^−1^ h^−1^ and was robust across the tested substrate mixtures.

#### Proof of concept: high‐cell‐density tailor‐made PHA production at pilot scale

**FIGURE 5:**
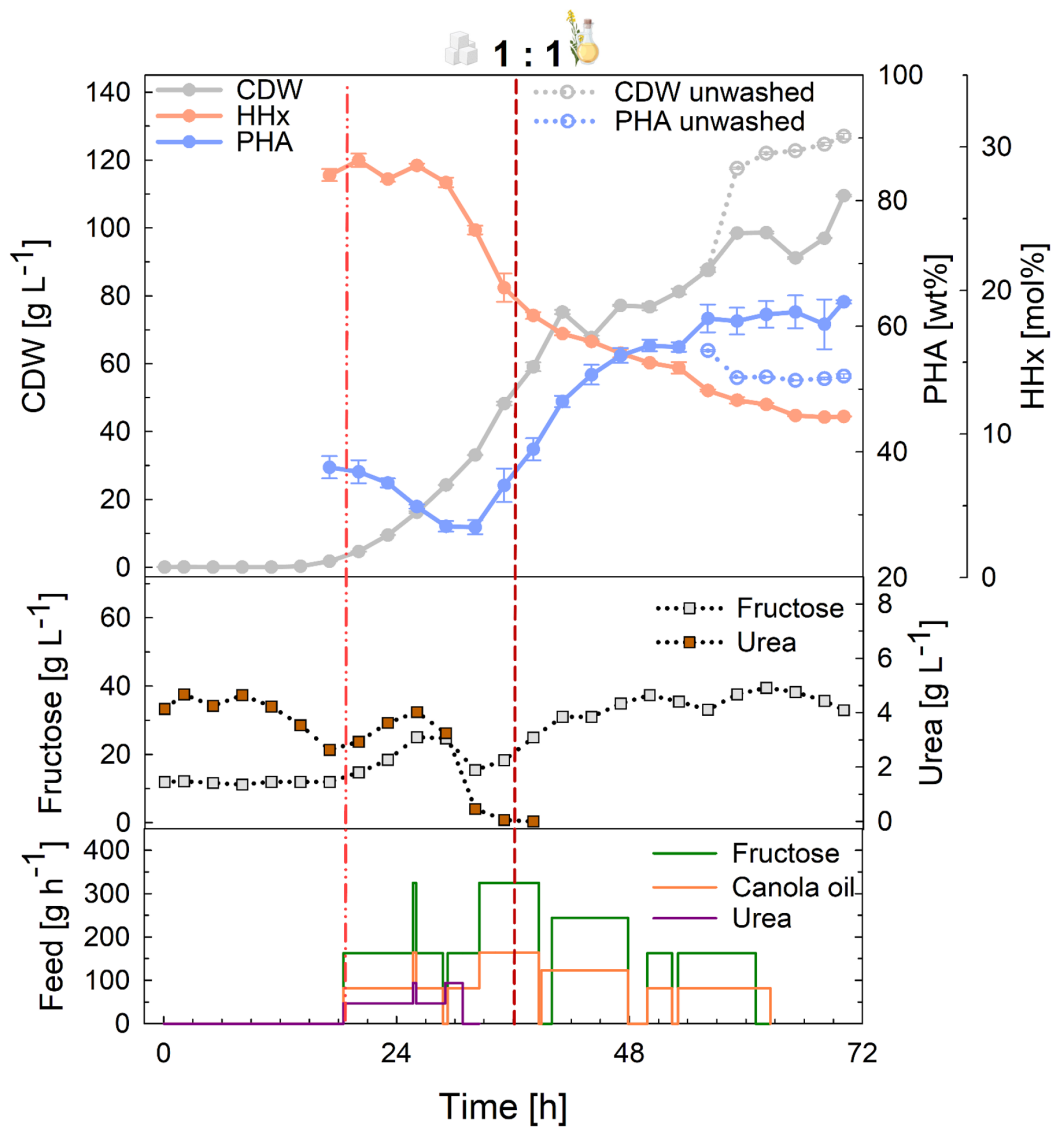
Tailored P(HB‐*co*‐HHx) production in high‐cell‐density fed‐batch mode at pilot scale using *R. eutropha* Re2058/pCB113 with a 1:1 mixture of fructose and canola oil as carbon sources and urea as nitrogen source. Cell dry weight (CDW, g L^−1^), PHA content of CDW (PHA, wt%), HHx content of PHA (HHx, mol%), fructose concentration (g L^−1^), urea concentration (urea, g L^−1^), and fructose, canola oil, and urea feeding rates (feed, g h^−1^) over the whole process time are shown. The final carbon content of the cultivation was 138 g L^−1^, and a C/N ratio of 22 g g^−1^ was used. Error bars represent the standard deviation of technical duplicates. Dashed lines indicate the feeding start (light red, dash–dot–dot) and nitrogen limitation (dark red, short dash).

Finally, the key objective was to scale the high‐cell‐density cultivation with the 1:1 fructose and canola oil mixture to pilot scale (Figure 5). Again, higher pump rates allowed for a more precise control of the substrate concentrations with less disturbances. As in previous cultivations, an interphase was visible when samples from the production phase were washed with *n*‐hexane and ice‐cold water that might correspond to lysed cells or very oily cells (see Figure S1). To address this, samples of the last 15 h before harvest were analysed, washed as well as unwashed. No difference in the final PHA titre was observed with final washed biomass values of 109 g L^−1^ containing 63.9 wt% PHA with an HHx content of 11.2 mol%, whereas the unwashed biomass yielded 128.7 g L^−1^ with 56.3 wt% PHA and 12.4 mol% HHx. At the end of the cultivation, after 72 h, no distinct oleaginous layer was observed on top of the centrifuged samples, indicating that the oil supplied was fully consumed. Residual fructose in the bioreactor that remained unconsumed was measured at a concentration of 33 g L^−1^.

### Scale‐up and productivity optimization of *R. eutropha* cultivations with tailored P(HB‐*co*‐HHx) compositions

**FIGURE 6:**
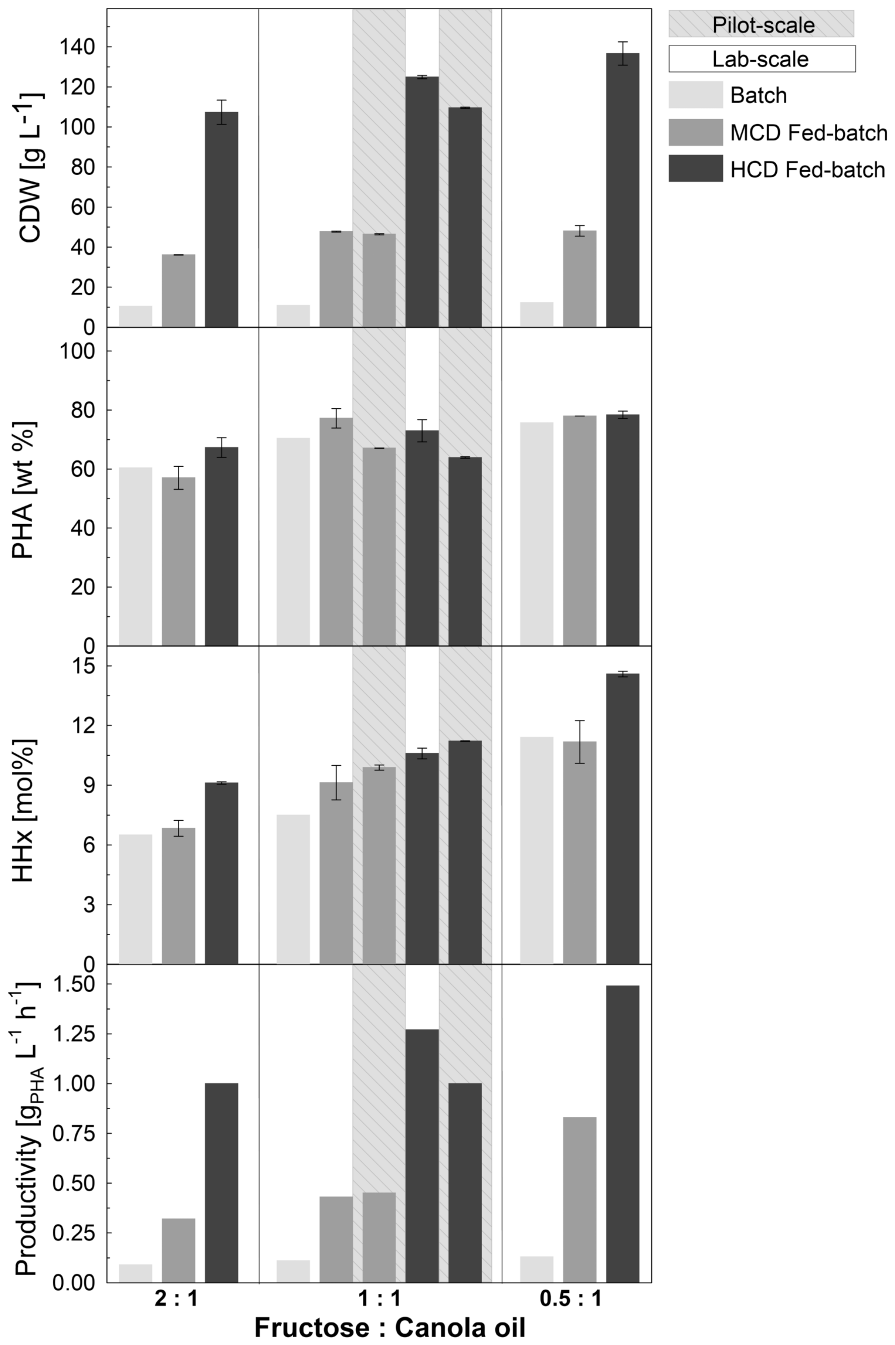
Overview of final yields. Cell dry weight (CDW; g L^−1^), PHA content of CDW (PHA; wt%), molar HHx fraction (HHx; mol%), and productivity (g_PHA_ L^−1^ h^−1^) across batch, medium‐cell‐density (MCD) fed‐batch, and high‐cell‐density (HCD) fed‐batch in 1‐L laboratory scale and 150‐L pilot scale of *R. eutropha* Re2058/pCB113 cultivations using substrate mixtures of fructose and canola oil at a ratio of 2:1, 1:1, and 0.5:1 (g g^−1^ carbon).

P(HB‐*co*‐HHx) compositions could be tailored by applying a linear feed of defined mixtures of fructose and canola oil yielding reproducible results across all tested cultivation strategies and scales (Figure 6, Table 1). Initial fed‐batch cultivations yielded tailor‐made P(HB‐*co*‐HHx) in the range of 6.8–11.2 mol% HHx, the same range previously demonstrated with these substrate mixtures in batch mode. High‐cell‐density cultivations showed slightly higher HHx contents in the range of 9.0–14.6 mol%. Comparing the results of the 1:1 fructose and canola oil mixtures, it can be observed that, while at pilot scale slightly lower CDWs with lower PHA contents were reached, the PHA produced had a slightly higher HHx content than at laboratory scale. As a result of this feeding strategy, the productivity was drastically increased from around 0.1 g_PHA_ L^−1^ h^−1^ for the previously published batch cultivations up to 1.5 g_PHA_ L^−1^ h^−1^ for the 0.5:1 fructose and canola oil mixture at laboratory scale (Figure 6, Table 1). At this scale, a general productivity increase by factor 12 could be achieved for each substrate mixture. Even at pilot scale, productivities reached 1 g_PHA_ L^−1^ h^−1^, making the process more technically relevant while still having the ability to steer the molar composition of the copolymer.

**TABLE 1:** Overview of *R. eutropha* Re2058/pCB113 cultivations to produce tailor‐made P(HB‐*co*‐HHx) at laboratory to pilot scale.

Scale	Mode	Fructose: canola oil ratio	Time (h)	CDW (g L^−1^)	PHA (wt%)	HHx (mol%)	Productivity (g_PHA_ L^−1^ h^−1^)	
Laboratory scale	Batch	2:1	72	10.5	60.40	6.50	0.09	Santolin et al. (2023)
		1:1	72	10.9	70.40	7.50	0.11	
		0.5:1	72	12.3	75.70	11.40	0.13	
	Fed‐batch	2:1	64	36.14 ± 0.12	57.03 ± 3.89	6.84 ± 0.40	0.32	This study
		1:1	60^a^	47.76 ± 0.24	77.20 ± 3.29	9.13 ± 0.86	0.61	
		0.5:1	45	48.08 ± 1.64	77.97 ± 0.01	11.17 ± 1.07	0.83	
	HCD fed‐batch	2:1	72	107.30 ± 6.03	70.65 ± 0.00	9.04 ± 0.00	1.05	
		1:1	72	124.88 ± 0.83	73.00 ± 3.79	10.60 ± 0.27	1.27	
		0.5:1	72	136.62 ± 5.83	78.39 ± 1.26	14.59 ± 0.14	1.49	
Pilot scale	Fed‐batch	1:1	69	46.43 ± 0.30	67.10 ± 0.07	9.89 ± 0.13	0.45	
	HCD fed‐batch	1:1	70	109.52^b^ ± 0.30	63.91 ± 0.3	11.22 ± 0.02	1.00	
		1:1	70	127.06^c^ ± 0.79	56.34 ± 0.64	12.39 ± 0.04	1.04	

*Note*: Scale (laboratory or pilot), cultivation mode (batch, fed‐batch, or high‐cell‐density fed‐batch) (HCD fed‐batch), applied ratio of fructose to canola oil (g g^−1^), process time (Time, h), cell dry weight (CDW, g L^−1^), PHA content of CDW (PHA, wt%), HHx content of PHA (HHx, mol%), and productivity (g_PHA_ L^−1^ h^−1^) are indicated for each cultivation. For laboratory scale cultivations, standard deviation of biological duplicates is indicated; for pilot scale cultivations, standard deviation of technical duplicates is indicated.

^a^ 26 h corresponding to the longer lag phase due to problems with the preculture were subtracted as a time correction for better comparability.

^b^ Big interphases occurred during *n*‐hexane wash step during CDW analysis (see Figure S1).

^c^ Cells were not washed with *n*‐hexane for CDW determination.

## DISCUSSION

In an effort to advance the biological production of PHA bioplastics, we demonstrated the laboratory to pilot scale high‐cell‐density production of tailor‐made P(HB‐*co*‐HHx) reaching productivities up to 1.5 g_PHA_ L^−1^ h^−1^ and confirming the viability and scalability of our substrate‐flexible strategy with *R. eutropha* Re2058/pCB113 to produce PHAs for versatile applications in the kilogram range at an industrially relevant scale.

In our previously published study (Santolin et al., 2023), we developed a batch strategy using mixtures of fructose and canola oil to tailor‐made HHx monomer content of P(HB‐*co*‐HHx) in the range of 2–17 mol% in 1‐L batch cultivations. The HHx monomer range achieved, which is limited in our strategy by the maximum amount obtained with canola oil as a pure substrate that contains 90% oleic acid (C18) (Saad et al., 2021), is narrow in comparison with other studies employing oleaginous feedstocks containing shorter fatty acids such as palm olein or date seed oil (Murugan et al., 2016; Purama et al., 2018). Nevertheless, this specific range (up to around 17 mol% HHx) has proven to be exceptionally compelling (Thiele et al., 2024), having already found commercial success through 4 companies: Danimer Scientific, Kaneka, Bluepha, and RWDC Industries, which commercialize them for diverse applications including shopping bags, cutlery, containers, food packaging, textiles, and adhesive coatings (Koller & Mukherjee, 2022; Tang et al., 2022). Characterization of the material properties depending on the HHx fraction ranging from 2 to 14 mol% revealed significant changes in both thermal and mechanical properties leading to a broadening of the processing window and application scope. Increase of the molar HHx content results in a decrease in both the melting temperature (*T*_m_) and the glass transition temperature (*T*_g_) of the copolymers, from *T*_m_ 165°C to 126°C and *T*_g_ 4°C to −5.9°C, respectively. With a molecular weight range between 3.2 and 4.0 × 10^5^ Da and a copolymer structure displaying a random distribution, crystallinity decreased from 54% to 20%. The elongation at break markedly improved, escalating from 5.7% to 703% at an HHx content of 14 mol%. This broad HHx content spectrum enabled the production of both ductile and brittle copolymers, all while preserving tensile strength throughout the examined range. Moreover, an increase in the HHx fraction was observed to decelerate abiotic degradation, diminishing it from 70% to 55% over a span of 12 h (Thiele et al., 2024). Still, in order to be industrially relevant, PHA production strategies need to be highly productive to guarantee low production prices and hence higher commercialization potential.

The impact of different substrate ratios on P(HB‐*co*‐HHx) compositions has also been shown for other recombinant strains of *R. eutropha* (Sato et al., 2015), and some approaches utilize genetic engineering to achieve specific P(HB‐*co*‐HHx) compositions (Arikawa & Matsumoto, 2016; Arikawa & Sato, 2022; Insomphun et al., 2015; Mifune et al., 2008, 2010) (Table 2). Apart from Arikawa and Matsumoto (2016), Arikawa and Sato (2022), and Sato et al. (2015) who demonstrated the production of tailor‐made P(HB‐*co*‐HHx) with exceptional productivities over 2 g_PHA_ L^−1^ h^−1^ by controlling *phaJ* expression in *R. eutropha* recombinant strains and by feeding a recombinant strain of *R. eutropha* with mixtures of butyrate and palm kernel oil, respectively, all previously published studies, including ours, only were able to tailor P(HB‐*co*‐HHx) compositions in low‐cell‐density cultivations with productivities below 0.2 g_PHA_ L^−1^ h^−1^. Notably, this study represents a significant advancement, achieving productivities of up to 1.5 g tailor‐made P(HB‐*co*‐HHx) L^−1^ h^−1^ in high‐cell‐density fed‐batch cultivations and rank, with about 1 g L^−1^ h^−1^, among the highest reported for any strain of *R. eutropha* at pilot scale. Other studies (Kahar et al., 2004; Riedel et al., 2012; Santolin et al., 2021; Sato et al., 2013; Thinagaran & Sudesh, 2017; Zainab‐L & Sudesh, 2019) also showed highly productive cultivations of *R. eutropha* strains achieving up to 1.2 g_PHA_ L^−1^ h^−1^, albeit without control over monomer compositions and only at maximum 6 L working volume.

**TABLE 2:** Comparison of productivities of shake flask and laboratory and pilot scale bioreactor cultivations using *Ralstonia eutropha* strains to produce P(HB‐*co*‐HHx) with tailored HHx contents.

Strain	Carbon source	HHx range (mol% HHx)	Productivity (g_PHA_ L^−1^ h^−1^)	Scale	Reference
Re2058/pCB113	Fructose and canola oil	7–15	0.32–1.49 0.45–1.04	Laboratory scale Pilot scale	This study
Re2058/pCB113	Fructose and canola oil	2–17	0.07–0.16	Laboratory scale	Santolin et al. (2023)
Re2058/pCB113	Palm olein and fructose	5–28	0.02–0.12	Laboratory scale	Murugan et al. (2017)
Re2058/pCB113	Crude palm kernel oil and oil palm tree trunk sap	14–27	0.03–0.09	Shake flask	Murugan et al. (2016)
Re2058/pCB113	Date seed oil and date molasses	2–39	<0.01–0.07	Shake flask	Purama et al. (2018)
β‐ketothiolase deletion strains	Palm kernel oil	14–32	2.64–2.88	Laboratory scale	Arikawa and Sato (2022)
*phaJ* expression‐controlled rec. strains	Palm kernel oil	6–15	2.49–2.57	Laboratory scale	Arikawa and Matsumoto (2016)
ASBK	Butyrate and palm kernel oil	7–13	1.74–2.12	Laboratory scale	Sato et al. (2015)
*phaC*_*Ac*_ in vitro evolution of rec. strains	Soya bean oil	3–10	0.05–0.07	Shake flask	Mifune et al. (2010)
*phaC*_*Ac*_ rec. strains	Octanoate	4–13	0.05–0.06	Shake flask	Mifune et al. (2008)
*ΔphaB1 Ccr* rec. strains	Fructose	0–22	<0.01–0.01	Shake flask	Insomphun et al. (2015)

To the best of our knowledge, this is the first study in the published literature that demonstrates the production of tailor‐made P(HB‐*co*‐HHx) with *R. eutropha* at an industrially relevant scale. It is important to note that, to date, only Ryu et al. (1997), who attained productivities of 3.1 g_PHB_ L^−1^ h^−1^ using a mutated *R. eutropha* strain able to utilize glucose and phosphate limitation to trigger PHB accumulation, could show higher productivities at pilot scale, while only producing the homopolymer PHB.

Although current trends underline the potential of using waste streams as cheap carbon sources for the production of PHAs (Brigham & Riedel, 2019; Che et al., 2023; Mahato et al., 2023), most companies that currently commercialize PHA still base their production on first‐generation renewable raw substrates such as virgin vegetable oils or sugars due to the inherent challenge of managing waste streams and coping with fluctuating substrate compositions (Koller & Mukherjee, 2022). Our strategy, although based on pure substrates, offers substrate flexibility to some extent, making it possible to imagine a scenario where copolymers with higher HHx titres are produced when canola oil or other plant oil prices drop, for example, during the harvest season (Gutschmann et al., 2022).

In this study, cells accumulated lower contents of PHA when mixtures with higher fructose ratios were fed, although the final carbon content provided with each mixture was constant, which was also reported in our previous study (Santolin et al., 2023). Higher dilutions caused by the fructose feed in contrast to pure canola oil and also the less efficient utilization of fructose by the strain that releases one molecule of CO_2_ for the conversion of each acetyl‐CoA by the pyruvate‐dehydrogenase are responsible for this effect. Furthermore, towards the end of the cultivation PHA contents did not increase although fructose accumulated in the media. The reason for this phenomenon is unclear but might be related to the expression of the ABC transporter for the sugar (frcABC), which is probably regulated by the transcriptional regulator FrcR (Kaddor & Steinbüchel, 2011). As shown in our and other previous studies (Murugan et al., 2017; Santolin et al., 2023), it is assumed that whereas canola oil is the preferred carbon source (fructose is not consumed during the initial cultivation period although biomass is being formed), the strain is later able to assimilate both fructose and canola oil simultaneously as no diauxic shift is visible in the dissolved oxygen signal suggesting a smooth transition (Figure S2).

Moreover, the superiority of plant oils as a substrate for PHA production with *R. eutropha* is apparent by the faster growth after the lag phase with sooner onset of nitrogen depletion and PHA accumulation to higher content of up to 78 wt%. This is reflected by productivities up to three times higher when comparing 0.5:1 to the 2:1 fructose and canola oil ratios in initial fed‐batch cultivations (Table 1).

The trend showing higher HHx molar contents when moving to higher‐cell‐density cultivations may be related to higher residual fructose concentrations left unconsumed during the cultivations with higher final carbon contents. This would nevertheless not explain the higher HHx content obtained with the 0.5:1 fructose and canola oil mixtures as fructose was depleted at the end of both cultivations.

Scalability from laboratory scale to pilot scale was overall simple and reproducible, while a further improvement of the productivity at pilot scale is possible by increasing the inoculation volume at this scale, which was only ~6% in comparison with the 10% utilized at laboratory scale. When upscaling, the feeding procedure was even simplified as the pump rates were more accurate with the pumps not running at their lower limit. Nevertheless, in larger scales, under long process times, back pressure from the reactor might demand a balance‐controlled feeding to accurately control the process. Further issues arising with an increasing scale are critical levels of foam and the high oxygen demand during the growth phase of a fed batch that is conducted with a substrate excess as beneficial for this process. To tackle these issues, a foam centrifuge was proven successful at pilot scale, while the oxygen demand was met by increasing the volumetric flow of air (Figure S2). Proceeding to even larger scales, where the power input is a limiting factor, addition of pure oxygen to the aeration and cultivating under overpressure can prevent oxygen limitation that was shown to be a trigger of PHA production (Blunt et al., 2019; Senior et al., 1972). At industrial scale, inhomogeneities and gradients form due to increased mixing times and a lower power input. Scale‐down studies could further be conducted to understand the effect of these gradients on the physiological state of the cells and on the resulting product yields (Neubauer & Junne, 2010).

## CONCLUSION AND OUTLOOK

We could demonstrate the optimization and scale‐up of our substrate‐flexible strategy with *R. eutropha* Re2058/pCB113 to produce up to 1.5 g L^−1^ h^−1^ P(HB‐*co*‐HHx) with tailored HHx compositions in the range between 7 and 15 mol%. This study marks a valuable milestone as we are the first to demonstrate the production of tailor‐made P(HB‐*co*‐HHx) with any *R. eutropha* strain at an industrial relevant scale (150‐L pilot scale) delivering PHA bioplastics with defined properties in the kilogram scale. By doing so, our study addresses the need for large polymer quantities required for testing additives and optimizing the machinery and process conditions of manufacturing processes, thereby contributing to the acceleration of PHA commercialization. We are confident that our process can be further scaled up on the path to commercial demonstration at the m^3^ scale. Moreover, our feeding strategy should be applicable to other native vegetable oils or used cooking oils with little or no adaption, which would increase substrate flexibility.

## AUTHOR CONTRIBUTIONS

**Isabel Thiele:** Conceptualization; methodology; data curation; investigation; validation; formal analysis; visualization; writing – original draft; writing – review and editing. **Lara Santolin:** Conceptualization; methodology; data curation; investigation; validation; formal analysis; writing – original draft; writing – review and editing. **Svea Detels:** Data curation; validation. **Riccardo Osele:** Data curation; validation. **Peter Neubauer:** Resources; writing – review and editing. **Sebastian L. Riedel:** Conceptualization; methodology; data curation; investigation; validation; supervision; funding acquisition; project administration; writing – review and editing.

## FUNDING INFORMATION

This research was supported by the German Federal Ministry of Education and Research, grant number 031B0833A.

## CONFLICT OF INTEREST STATEMENT

The authors declare no conflict of interest.

## Supporting information


Appendix S1

## Data Availability

The data that support the findings of this study are available from the corresponding author upon reasonable request.
