# Supplementary material for: High‐cell‐density fed‐batch strategy to manufacture tailor‐made P(HB‐ co ‐HHx) by engineered Ralstonia eutropha at laboratory scale and pilot scale
Source: Microb Biotechnol. 2024 Jun 8;17(6):e14488. doi: 10.1111/1751-7915.14488 (PMC11162103; doi:10.1111/1751-7915.14488)
Supplement: Supplementary file 1 — Appendix S1 [file MBT2-17-e14488-s001.docx]

# Appendix/Supplementary

#
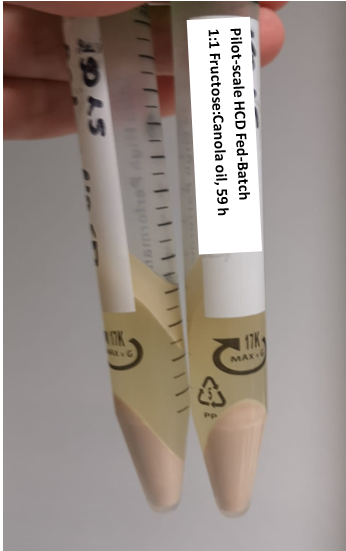


**Figure S1 |** Exemplary photo of the interphase obtained after washing with n-hexane and water and centrifugation of a sample of a pilot-scale cultivation of R. eutropha Re2058/pCB113 using a 1:1 mixture of fructose and canola oil. Sample after 59 h is shown. The interphase might be caused due to lysed cells or fatty acids disturbing the cellular membranes.


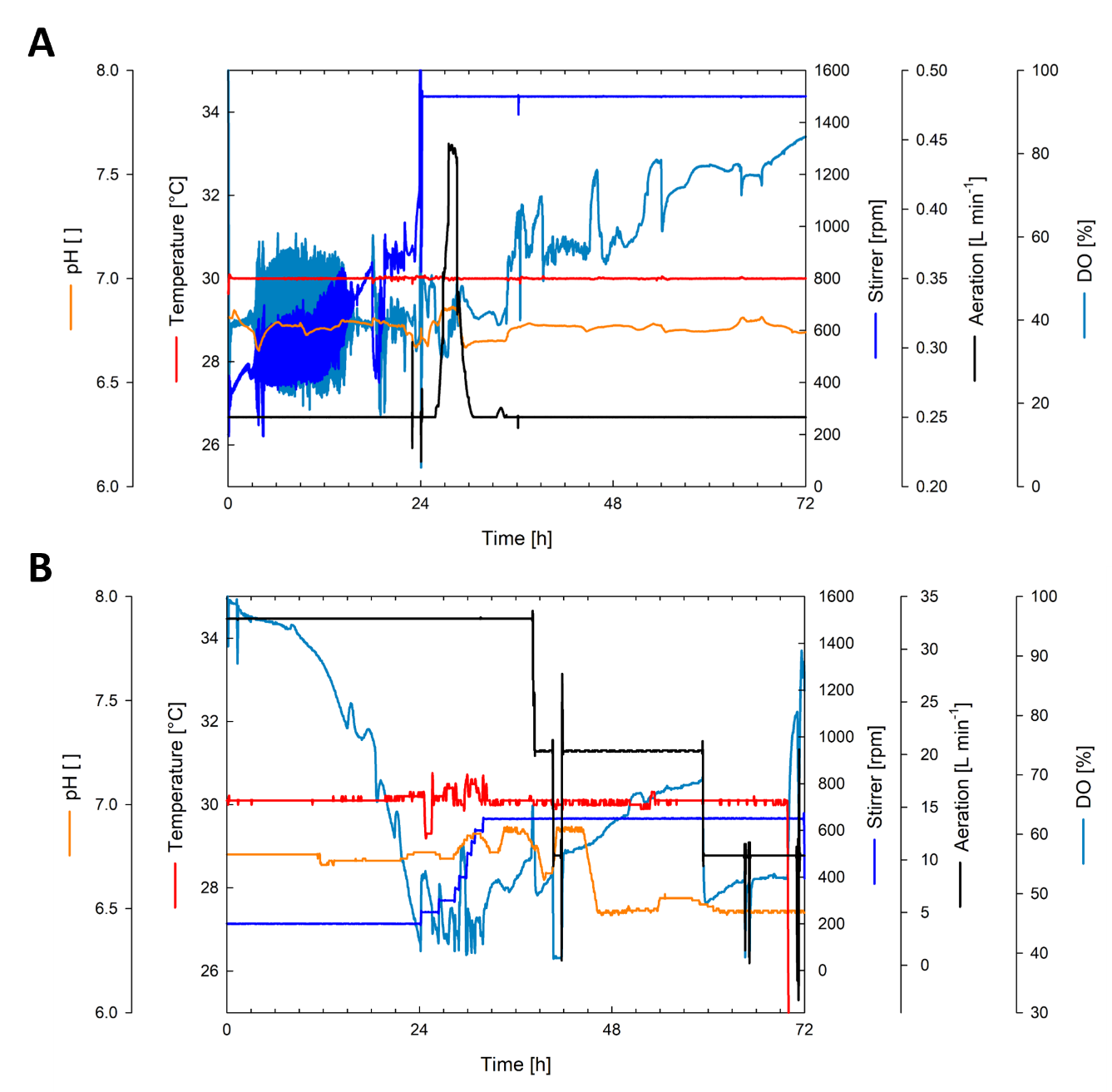


**Figure S2 |** Process data during laboratory- (A) and pilot-scale (B) cultivations in high-cell-density fed-batch mode using R. eutropha Re2058/pCB113 with a 1:1 mixture of fructose and canola oil as carbon sources and urea as nitrogen source.
